# Prediction and Analysis of Antibody Amyloidogenesis from Sequences

**DOI:** 10.1371/journal.pone.0053235

**Published:** 2013-01-07

**Authors:** Chyn Liaw, Chun-Wei Tung, Shinn-Ying Ho

**Affiliations:** 1 Institute of Bioinformatics and Systems Biology, National Chiao Tung University, Hsinchu, Taiwan; 2 School of Pharmacy, College of Pharmacy, Kaohsiung Medical University, Kaohsiung, Taiwan; 3 Department of Biological Science and Technology, National Chiao Tung University, Hsinchu, Taiwan; Center for Genomic Regulation, Spain

## Abstract

Antibody amyloidogenesis is the aggregation of soluble proteins into amyloid fibrils that is one of major causes of the failures of humanized antibodies. The prediction and prevention of antibody amyloidogenesis are helpful for restoring and enhancing therapeutic effects. Due to a large number of possible germlines, the existing method is not practical to predict sequences of novel germlines, which establishes individual models for each known germline. This study proposes a first automatic and across-germline prediction method (named AbAmyloid) capable of predicting antibody amyloidogenesis from sequences. Since the amyloidogenesis is determined by a whole sequence of an antibody rather than germline-dependent properties such as mutated residues, this study assess three types of germline-independent sequence features (amino acid composition, dipeptide composition and physicochemical properties). AbAmyloid using a Random Forests classifier with dipeptide composition performs well on a data set of 12 germlines. The within- and across-germline prediction accuracies are 83.10% and 83.33% using Jackknife tests, respectively, and the novel-germline prediction accuracy using a leave-one-germline-out test is 72.22%. A thorough analysis of sequence features is conducted to identify informative properties for further providing insights to antibody amyloidogenesis. Some identified informative physicochemical properties are amphiphilicity, hydrophobicity, reverse turn, helical structure, isoelectric point, net charge, mutability, coil, turn, linker, nuclear protein, etc. Additionally, the numbers of ubiquitylation sites in amyloidogenic and non-amyloidogenic antibodies are found to be significantly different. It reveals that antibodies less likely to be ubiquitylated tend to be amyloidogenic. The method AbAmyloid capable of automatically predicting antibody amyloidogenesis of novel germlines is implemented as a publicly available web server at http://iclab.life.nctu.edu.tw/abamyloid.

## Introduction

Antibody-based therapy characterized by its high specificity to targeted antigens has been adopted for treatments of cancer, autoimmune disease and inflammatory. Monoclonal antibodies are usually derived from murine and suffer from short half-life and undesirable immunogenicity that largely reduce the therapeutic effects [1], [2]. Humanized antibodies are developed to overcome the above-mentioned problems by grafting murine variable domains, the specificity-determining residues, and complementarity-determining residues [3], [4], [5], [6]. However, the humanization process might decrease thermal stability of antibodies that could affect their affinities to targets and lead to amyloid fibril formation [3], [7], [8]. The experiments for humanizing antibodies are expensive and time-consuming. Consequently, it is desirable to develop an accurate method for predicting antibody amyloidogenesis.

Several important properties have been found to be related to amyloidogenesis. Alternating patterns of polar/hydrophilic and nonpolar/hydrophobic amino acids promote amyloid-like structures [9], [10], [11], [12]. Amphiphilicity is found to be important in determining the beta-sheet structure of amyloid fibrils [13] and is a common property of amyloids [14], [15]. Hydrophobicity is correlated with aggregation [16], [17], [18], [19], [20]. Reverse turn is an important feature of the Alzheimer’s *β*-amyloid protein [21], [22]. The helical structure of intermediates is important for amyloid formation [23], [24]. For the isoelectric point, *β*-lactoglobulin aggregates into spherical aggregates at the isoelectric point and amyloid fibril away from the isoelectric point [25]. The change of net charge is also found to be correlated with protein aggregation [10], [17], [26].

Previous studies show that fibril formation is influenced by the sequence and stability of proteins [27], [28], [29], [30]. The correlation between sequence variations and amyloidogenesis enables the sequence-based prediction of antibody amyloidogenesis. Several studies were proposed to predict amyloidogenic potential of polypeptides [31], [32], [33], [34] and amyloidogenic regions [21], [35], [36], [37], [38], [39], [40]. However, they are not able to give a clear prediction on whether a humanized antibody will be amyloidogenic or not.

Recently, a computational method was proposed to predict amyloidogenesis in antibodies by using Naïve Bayes classifiers and decision tree methods with reasonably high prediction accuracies [41]. However, there are two shortcomings in utilizing this method [41]. (1) The necessary alignment procedure needs experts to manually adjust the alignment. Also, the decision trees of their study are manually designed. (2) The germline-specific method establishes individual models for each known germline. Germlines represent the basic and inherited antibody repertoire of an individual. The derivative sequences are produced by rearranging and mutating the protein sequences of germlines during the response to foreign antigens. However, the antibody amyloidogenesis as a global property of an antibody should be germline-independent. Therefore, it is desirable to develop an alignment-free and across-germline method for automatically predicting antibody amyloidogenesis of novel germlines from sequences.

In this study, three types of germline-independent sequence features are used to encode antibody sequences including amino acid composition, dipeptide composition and physicochemical properties. The use of germline-independent sequence features enables analysis and prediction of antibody amyloidogenesis across germlines that is important to further applications for antibodies of novel germlines. The dipeptide composition feature performs best in all within-, across- and novel-germline predictions. A prediction method named AbAmyloid based on the Random Forests classifier and dipeptide composition feature is proposed.

For many existing germlines, there are not enough corresponding derivatives to design germline-dependent prediction methods at present. Because of lack of reference sequences, it is also difficult to predict amyloidogenesis of novel germlines. However, a method capable of predicting antibody amyloidogenesis of novel germlines can be helpful to antibody designs. AbAmyloid with an accuracy of 72.22% on 12 germlines using a leave-one-germline-out test is a potentially good prediction method of antibody amyloidogenesis for novel germlines. As the number of germlines in the training data sets increases, the prediction accuracy is expected to be higher from our results.

The feature importance is analyzed to provide good understanding of correlations between the germline-independent sequence features and antibody amyloidogenesis. Additionally, the predicted ubiquitylation sites by the UbiPred program [42] are found to be correlated with antibody amyloidogenesis that antibodies less likely to be ubiquitylated tend to be amyloidogenic.

## Results and Discussion

### Performance of Methods for Predicting Aggregation Prone Regions

Several studies were proposed to predict aggregation prone regions that include AGGRESCAN [32], PASTA [37], TANGO [40], etc. AGGRESCAN calculates the prediction score for aggregation “hot spot” using the aggregation-propensity values for each of the 20 amino acids derived from previously experimental data. TANGO is a statistical mechanics algorithm to predict β-aggregation propensities. PASTA calculates the energy of β-pairings to predict protein aggregation. We apply these three general methods to predict all sequences in the AA-432 dataset and calculate the prediction performance using the score for the most aggregation prone region. Performance comparison is shown in Table 1.

**Table 1:** Performance comparison of three general methods.

Method	Sensitivity	Specificity	Accuracy (%)	AUC
**AGGRESCAN ** [**32**]	1.000	0.000	56.94	0.528
**TANGO ** [**40**]	0.911	0.059	54.40	0.519
**PASTA ** [**37**]	0.886	0.156	57.18	0.612

The accuracy, sensitivity and specificity of PASTA are 57.18%, 0.886 and 0.156 using the default threshold value −4.0, respectively. The accuracy, sensitivity and specificity of AGGRESCAN using the score of the most aggregation prone region with the default threshold value −0.02 are 56.94%, 1.000 and 0.000, respectively. The accuracy, sensitivity and specificity of TANGO using the score of the most aggregation prone region with the default threshold value 5% are 54.40%, 0.911 and 0.059, respectively. The results indicate that the three methods for predicting aggregation prone regions get low values of specificity. These general methods trained using only amyloidogenic proteins aim to predict which regions of a sequence are potentially amyloidogenic. Therefore, it is not easy to distinguish between amyloidogenic and non-amyloidogenic antibodies with highly similar sequences [41].

**Figure 1:**
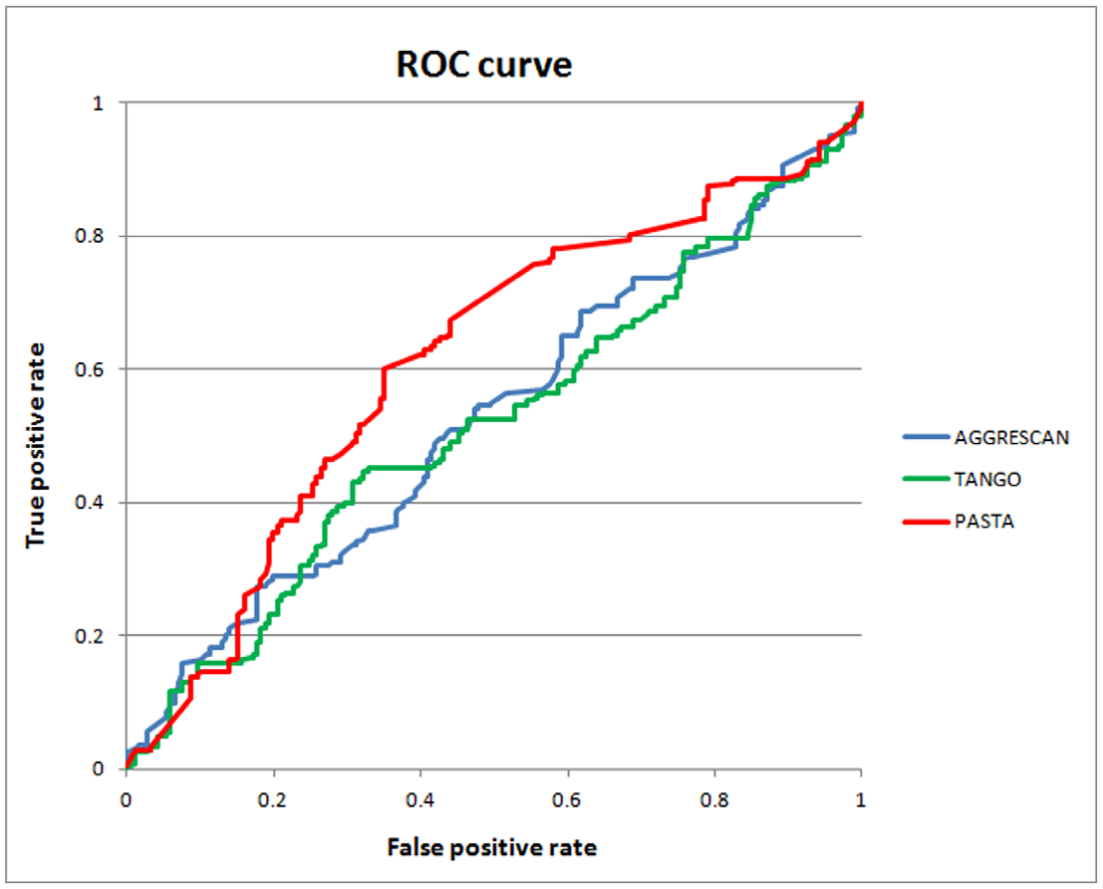
The receiver operator characteristic (ROC) curves of three general methods.

In addition, we normalize the scores of different methods into the numeric range of −1 to 1. Considering their different predictive thresholds, the Receiver Operator Characteristic (ROC) curve and area under the ROC curve (AUC) are used to evaluate the performance of all predictors that are shown in Figure 1 and Table 1. The AUCs of AGGRESCAN, TANGO and PASTA are 0.528, 0.519 and 0.612, respectively. The results indicate that some regions are prone to aggregation in both amyloidogenic and non-amyloidogenic sequences. However, it is still hard to determine whether the sequence is amyloidogenic or not.

### Within-germline Prediction of Antibody Amyloidogenesis

**Figure 2:**
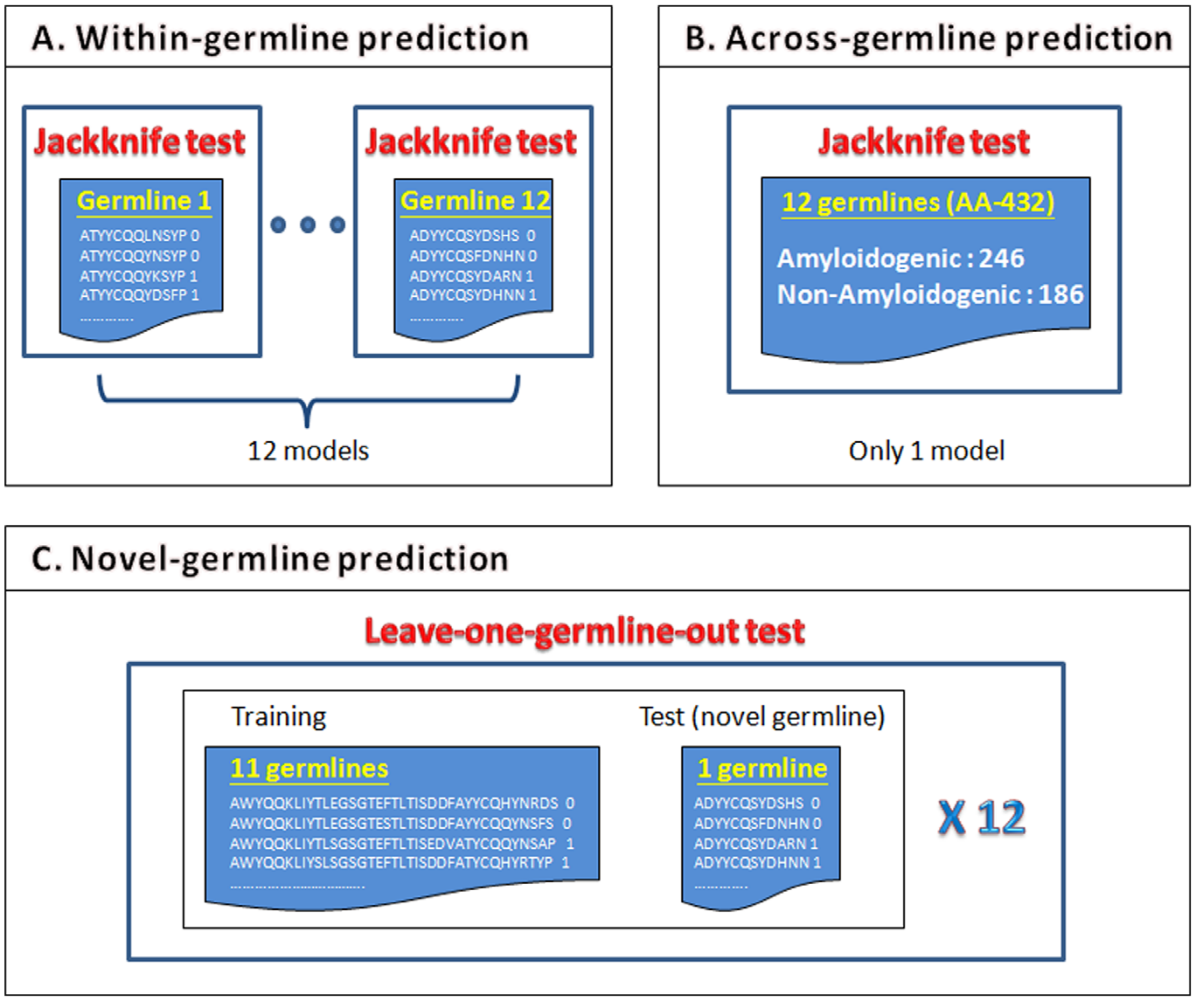
Three evaluation methods for AbAmyloid. (A) There are 12 individual germline models. Each model is evaluated using a Jackknife test. (B) Only one model is constructed using a dataset of 12 germline (AA-432). (C) The leave-one-germline-out test is applied to evaluate the novel-germline prediction of AbAmyloid where each dataset of one germline is served as the test dataset of novel germline in turn.

Informative features are critical for designing an accurate classifier and providing good understanding of antibody amyloidogenesis. Three types of germline-independent sequence features, amino acid composition (AAC), dipeptide composition (DPC) and physicochemical properties (PPs) are utilized to encode antibody sequences. An efficient Random Forests method is adopted to evaluate the three types of features and their combinations in terms of Jackknife test accuracy. All sequences of a given germline, except that one sequence used for independent test, are used to develop a germline model. Therefore, each germline dataset generates one independent model, shown in Figure 2(A). Performance comparison among various types of features and methods for the within-germline prediction using a Jackknife test is shown in Table 2. The best accuracy 83.10% of using the feature DPC shows superiority of DPC in predicting antibody amyloidogenesis. The other two features AAC and PPs with accuracies of 78.24% and 76.85%, respectively, perform worse than DPC.

**Table 2:** Performance comparison among various types of sequence features and methods for the within-germline prediction in terms of Jackknife test accuracy.

Method	Sensitivity	Specificity	Accuracy (%)
**Random Forests (AAC)**	0.813	0.742	78.24
**Random Forests (DPC)**	0.829	0.833	83.10
**Random Forests (PPs)**	0.825	0.694	76.85
**Random Forests (AAC+DPC)**	0.829	0.801	81.71
**Random Forests (AAC+PPs)**	0.850	0.720	79.40
**Random Forests (DPC+PPs)**	0.850	0.747	80.56
**Random Forests** **(AAC+DPC+PPs)**	0.850	0.753	80.79
**Naïve Bayes ** [**41**]	0.756	0.823	78.47

Using combinations of the three feature types might yield better prediction performance. Therefore, four combinations of feature types are also assessed. The highest accuracy of 81.71% is achieved by using the combination of AAC and DPC. The combination of AAC, DPC and PPs performs well with the second highest accuracy of 80.79%. The results show that DPC is highly relevant to predicting antibody amyloidogenesis. The overall accuracy of the DPC model for the 12 datasets is 83.10% which is better than the existing method of using Naïve Bayes with an accuracy of 78.47% [41]. Please note that the proposed method do not require manual alignment as the method [41] and can automatically predict antibody amyloidogenesis.

### Across-germline Prediction of Antibody Amyloidogenesis

In contrast to the existing germline-dependent method [41], this study proposes a germline-independent method which is capable of performing across-germline prediction of antibody amyloidogenesis without knowing which germline of the query sequence is in advance. To show the across-germline ability of predicting antibody amyloidogenesis, the Jackknife test performance on the dataset AA-432 consisting of 12 germlines is obtained for the three types of features. All sequences of all the 12 germlines, except that one sequence left out of the training dataset for independent test, are used to develop a general model. Each sequence of the dataset AA-432 is used as a test sequence in turn on the general model, as shown in Figure 2(B).

Performance comparison among various types of features and methods for the across-germline prediction is shown in Table 3. Similar to the within-germline prediction results, the feature DPC performs best with an accuracy of 83.33%. The other two features AAC and PPs perform slightly worse than DPC with accuracies of 79.63% and 79.17%, respectively. For the Jackknife test performances of four combinations of these feature types, the highest accuracy of 83.10% is achieved by using the combination of AAC and DPC. The combination of DPC and PPs performs well with the second highest accuracy of 82.87%. The accuracies of the best feature type (DPC) and the best two combinations of sequence features are nearly the same.

**Table 3:** Performance comparison among various types of sequence features and methods for the across-germline prediction in terms of Jackknife test accuracy.

Method	Sensitivity	Specificity	Accuracy (%)
**Random Forests (AAC)**	0.846	0.731	79.63
**Random Forests (DPC)**	0.854	0.806	83.33
**Random Forests (PPs)**	0.846	0.720	79.17
**Random Forests (AAC+DPC)**	0.854	0.801	83.10
**Random Forests (AAC+PPs)**	0.854	0.731	80.09
**Random Forests (DPC+PPs)**	0.870	0.774	82.87
**Random Forests** **(AAC+DPC+PPs)**	0.858	0.763	81.71

By comparing the performances of within-germline and across-germline models (Tables 2 and 3), all the across-germline prediction accuracies are slightly higher than those of within-germline predictions for the seven feature sets evaluated. The mean accuracy (81.414%) of across-germline prediction is also higher than that (80.093%) of within-germline prediction due to a larger training dataset used. These results agree that antibody amyloidogenesis is considered a property of a whole antibody sequence independent from the corresponding germline.

Additionally, the 10-fold cross-validation on the dataset AA-432 is performed where the ratios of positive to negative samples for training and validation (independent test) dataset are the same ratio. Notably, the validation dataset is not involved in training models. The results of 10-fold cross-validation are shown in Table 4. The feature DPC performs best with an accuracy of 84.49%. The other two features AAC and PPs with accuracies of 80.09% and 78.01%, respectively, perform worse than DPC. The results confirm that DPC is highly relevant to predicting antibody amyloidogenesis well.

**Table 4:** Performance comparison among various types of sequence features and methods for the across-germline prediction using 10-fold cross-validation.

Method	Sensitivity	Specificity	Accuracy (%)
**Random Forests (AAC)**	0.846	0.742	80.09
**Random Forests (DPC)**	0.862	0.823	84.49
**Random Forests (PPs)**	0.837	0.704	78.01
**Random Forests (AAC+DPC)**	0.870	0.801	84.03
**Random Forests (AAC+PPs)**	0.837	0.715	78.47
**Random Forests (DPC+PPs)**	0.862	0.780	82.64
**Random Forests** **(AAC+DPC+PPs)**	0.862	0.785	82.87

### Novel-germline Prediction of Antibody Amyloidogenesis

Because amyloidogenesis is a property of a whole antibody sequence independent from the corresponding germline, a useful method should be able to predict antibody amyloidogenesis of novel germlines that is impossible for the existing method [41] and is much harder than the within- and across-germline predictions. To evaluate AbAmyloid in predicting antibodies of novel germlines, an experiment of using a leave-one-germline-out test is performed as following. All sequences of all the 12 germlines, except that all sequences of one germline *g* are left out of the training dataset, are used to develop a general model. Notably, the novel-germline prediction method established this model considering the whole set of all 11 germlines but not individual germlines. For each *g* of the 12 germlines in turn, antibody sequences of the germline *g* are used as an independent test dataset, shown in Figure 2(C).

**Figure 3:**
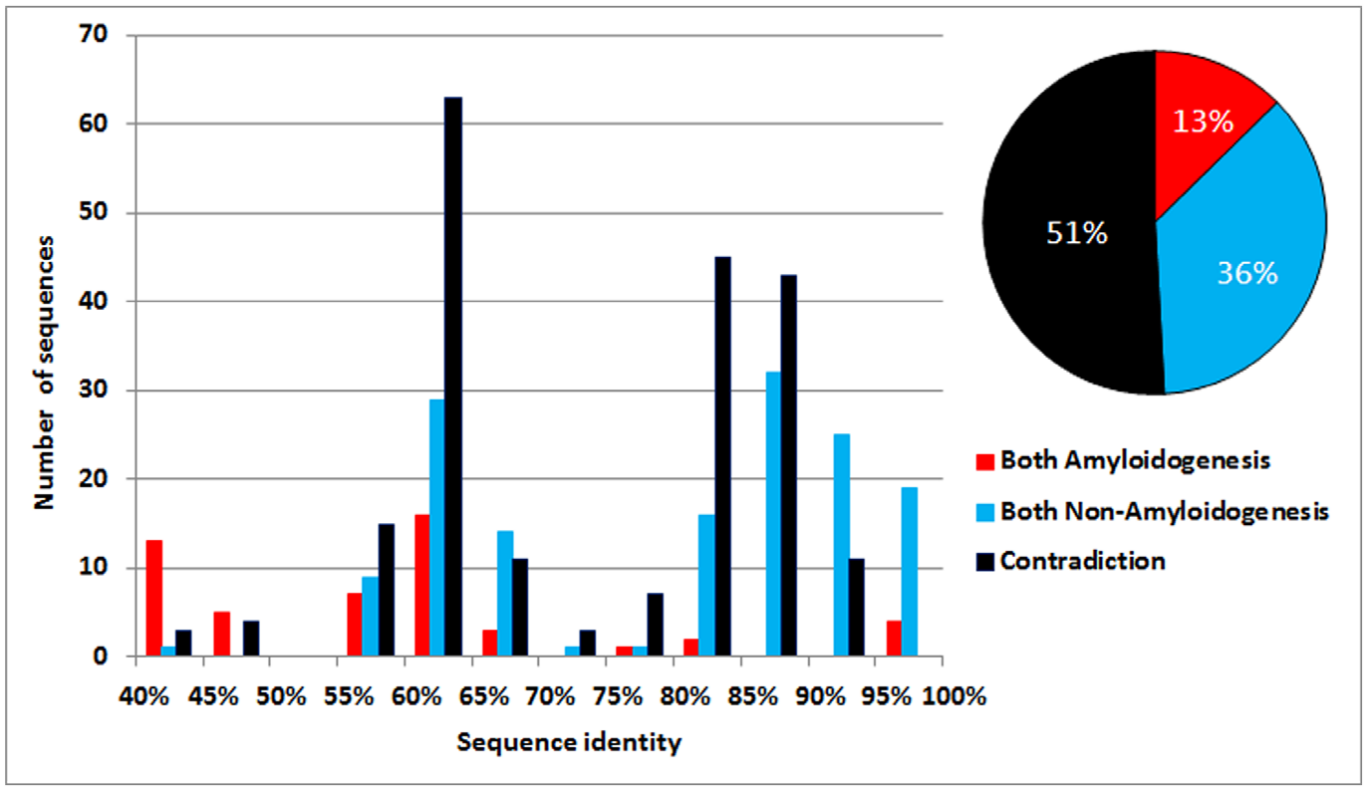
Histogram and percentages for sequence pairs with sequence identities between training and test datasets.

**Figure 4:**
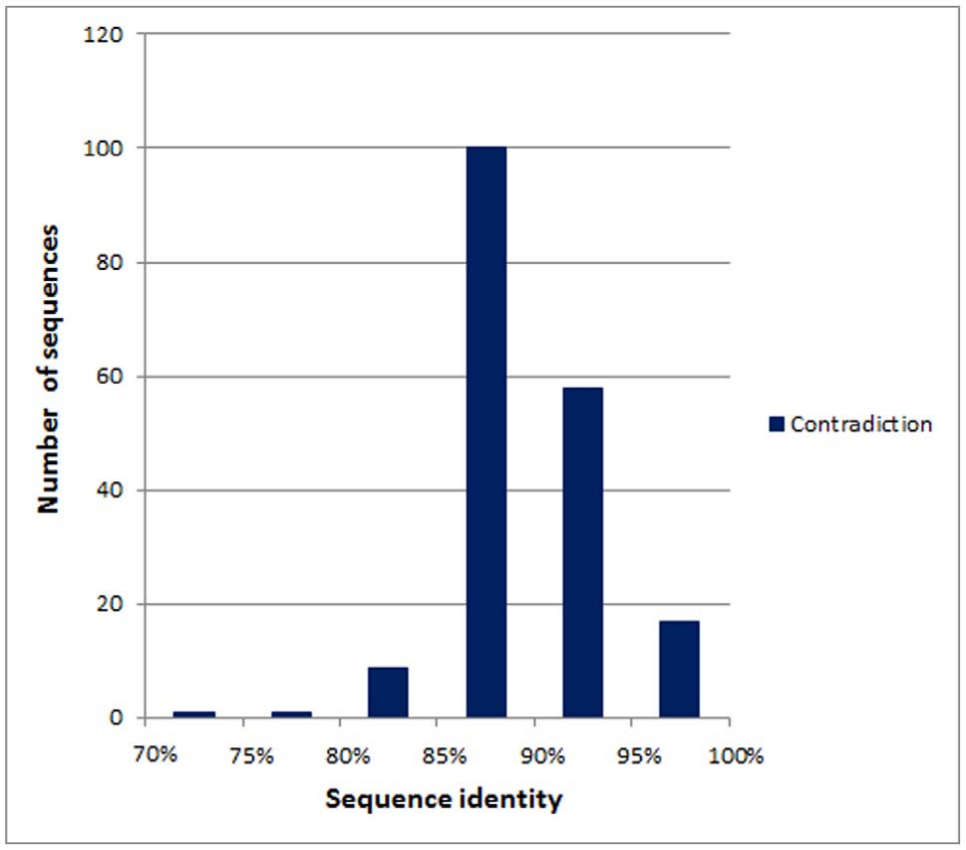
Histogram for sequence pairs with sequence identities between amyloidogenic and non-amyloidogenic sequences.

Sequences in the same germline are similar consisting of a protein sequence of germline and its derivatives that are rearranged and mutated from the protein sequence of germline with only a few changes of amino acids. We use CD-HIT-2D [43] to compare sequence identities between training and test datasets in the novel-germline prediction. The histogram and percentages for sequence pairs with sequence identities of both amyloidogenesis, both non-amyloidogenesis, and in contradiction are shown in Figure 3. The percentages of both amyloidogenesis, both non-amyloidogenesis, and in contradiction sequence pairs in all sequence pairs are 12.65%, 36.48% and 50.87%, respectively. The contradiction sequence pairs have the largest percentage in all sequence pairs. In the test datasets of leave-one-germline-out test, there are 29 sequences (7%) with sequence identity less than 40%, and the average sequence identity for the other sequences is 75% when comparing to the corresponding training datasets. However, the sequence identity between amyloidogenic and non-amyloidogenic sequences is 90% in the dataset AA-432. The histogram of sequence pairs with sequence identities between amyloidogenic and non-amyloidogenic sequences is shown in Figure 4. We also use BLAST [44] to calculate the prediction performance for novel-germline prediction. The accuracy, sensitivity and specificity of BLAST are 51.16%, 0.411 and 0.645, respectively. Therefore, it is hard to distinguish between amyloidogenic and non-amyloidogenic sequences based on sequence identity.

The prediction performances on the 12 germlines are shown in Table 5. The feature DPC with an overall accuracy of 72.22% is the best for the novel-germline prediction. The other two features AAC and PPs perform much worse than DPC with accuracies of 60.65% and 59.26%, respectively. The combination of AAC and DPC with an accuracy of 71.06% performs best, and the combination of DPC and PPs has the second highest accuracy of 68.75%. The results confirm the contribution of DPC in predicting antibody amyloidogenesis again. The sensitivity and specificity of using the feature DPC are 0.785 and 0.640, respectively. The benefit of high sensitivity/specificity is that most amyloidogenic/non-amyloidogenic sequences can be identified for experimental verification. The decision threshold of the Random Forests classifier for classification can be changed to adjust sensitivity and specificity according to preference. In order to consider the influence of sequence redundancy, we remove 4 duplicate sequences of non-amyloidogenic peptides among 3 germline datasets of AA-432 and recalculate the performance of DPC. The accuracy, sensitivity and specificity are 71.70%, 0.785 and 0.624 that are only slightly worse than the previous result, respectively.

**Table 5:** Performance comparison among various types of sequence features and methods for the novel-germline prediction.

Method	Sensitivity	Specificity	Accuracy (%)
**Random Forests (AAC)**	0.626	0.581	60.65
**Random Forests (DPC)**	0.785	0.640	72.22
**Random Forests (PPs)**	0.675	0.484	59.26
**Random Forests (AAC+DPC)**	0.768	0.634	71.06
**Random Forests (AAC+PPs)**	0.671	0.522	60.65
**Random Forests (DPC+PPs)**	0.732	0.629	68.75
**Random Forests** **(AAC+DPC+PPs)**	0.699	0.602	65.74
**BLAST ** [**44**]	0.411	0.645	51.16

As shown in Table 6, the detailed results of using DPC show that the highest two prediction accuracies of 94.05% and 81.40% are achieved by germlines Z73673 and Z22197, respectively. There are five and two germlines with accuracies larger than 70% and close to 70%, respectively. In contrast, two germlines X93632 and Z22188 with accuracies less than 40% suffer from improper selection of decision values for classification of amyloidogenic and non-amyloidogenic sequences where their sensitivity and specificity are 1.0 and 0, and 0.118 and 1.0, respectively. That is not comparable with their area under the receiver operating characteristic curves of larger than 0.7. Possible reasons for the variations of accuracy, sensitivity and specificity among germlines are that 1) the ratios of amyloidogenic and non-amyloidogenic sequences for each germlines are much different, and 2) the selection of a decision threshold for classification depends on the whole unbalanced training dataset but not individual germlines.

**Table 6:** The novel-germline prediction performances for 12 germlines using dipeptide composition (DPC).

Germline	Sensitivity	Specificity	Accuracy (%)
**J00248**	1.000	0.600	73.91
**M30446**	0.333	0.900	68.75
**X72813**	0.875	0.684	74.07
**X93620**	0.848	0.375	69.39
**X93627**	0.579	1.000	75.76
**X93632**	1.000	0.000	35.71
**X93640**	1.000	0.000	56.67
**Z22188**	0.118	1.000	34.78
**Z22191**	0.400	1.000	78.57
**Z22197**	0.962	0.588	81.40
**Z22208**	0.943	0.444	77.36
**Z73673**	1.000	0.853	94.05

An extensively used method based on support vector machine (SVM) is also utilized to evaluate the leave-one-germline-out test performance using the same DPC feature. The SVM method uses a radial basis function kernel with control parameters C and γselected by the grid search method [42] using 10-fold cross-validation on the training dataset. The test accuracy of SVM for the novel-germline prediction is 65.05%. The high performance of AbAmyloid with an accuracy of 72.22% may arise mainly from the used ensemble strategy, compared with the SVM method. Please note that prediction performance of the SVM method can be improved by further tuning the control parameters cooperated with an appropriate feature selection. This study utilizes the Random Forests method because of its built-in ability of feature importance estimation.

### Prediction Server AbAmyloid

The prediction method AbAmyloid uses a Random Forests classifier with DPC on the dataset AA-432 of 12 germlines. AbAmyloid performs well for predicting antibody amyloidogenesis with accuracies of 83.10%, 83.33% and 72.22% using the within-, across- and novel-germline predictions, respectively. The server of AbAmyloid with automatic amyloidogenesis prediction without knowing the germline name of query sequences in advance and prediction score ranged from 0 to 100% is publicly available at http://iclab.life.nctu.edu.tw/abamyloid.

**Figure 5:**
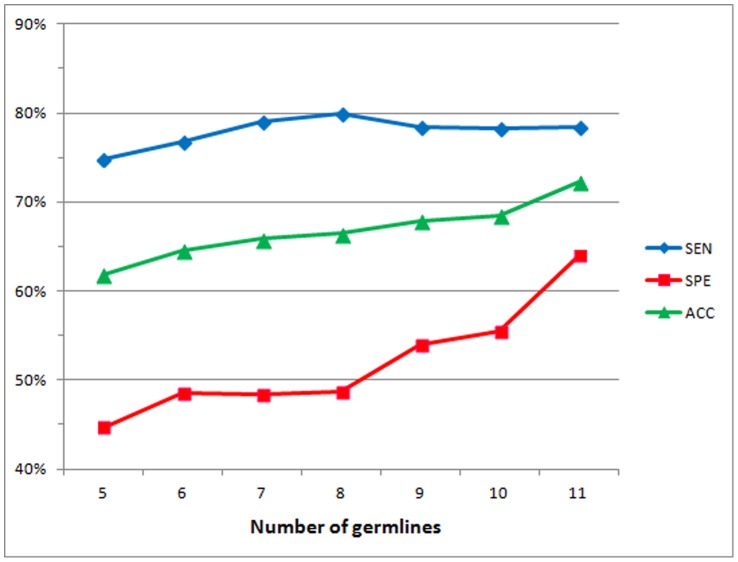
Learning curves using various numbers of germlines for training classifiers. The prediction performance is evaluated by using a leave-one-germline-out test.

The prediction performance might be improved by enlarging the training dataset. A learning curve experiment can be utilized to examine the correlation between dataset sizes and prediction accuracies. Based on the framework of leave-one-germline-out tests, 30 sets consisting of *n* germlines randomly selected are used to build training datasets where *n*∈{5, 6, …, 9}. Prediction accuracy for each fold is calculated by averaging the 30 results of predictions on the leaved germline dataset. For the training dataset consisting of 10 germlines, there are only 11 combinations. The average performance is calculated by using the 11 prediction results for each ford. Similarly, only one prediction result is obtained by using all 11 germlines for each ford. As shown in Figure 5, the prediction accuracy increases when there are more germlines in the training dataset. AbAmyloid is expected to be more accurate when more germlines in a larger dataset are available in building the prediction model.

In contrast to the existing method [41] relying on the manual sequence alignment for predicting sequences with known germlines, AbAmyloid capable of automatic prediction of antibody amyloidogenesis for novel germlines is more useful for further applications.

### Feature Importance of Composition Features

The analysis of feature importance for each type of sequence features can provide better understanding of antibody amyloidogenesis. The efficient and effective built-in feature importance estimator of the Random Forests method is applied to identify informative features for each feature type. Generally, two measures based on the mean decrease of Gini index (MDGI) and prediction accuracy are available for ranking feature importance. A recent study showed that the MDGI provides more robust results compared with the mean decrease of accuracy [45]. In this study, the MDGI is adopted to rank feature importance. To avoid the bias of random seed in evaluating feature importance, the average value of the MDGI on 30 runs of feature importance evaluation is used in the following analysis.

**Figure 6:**
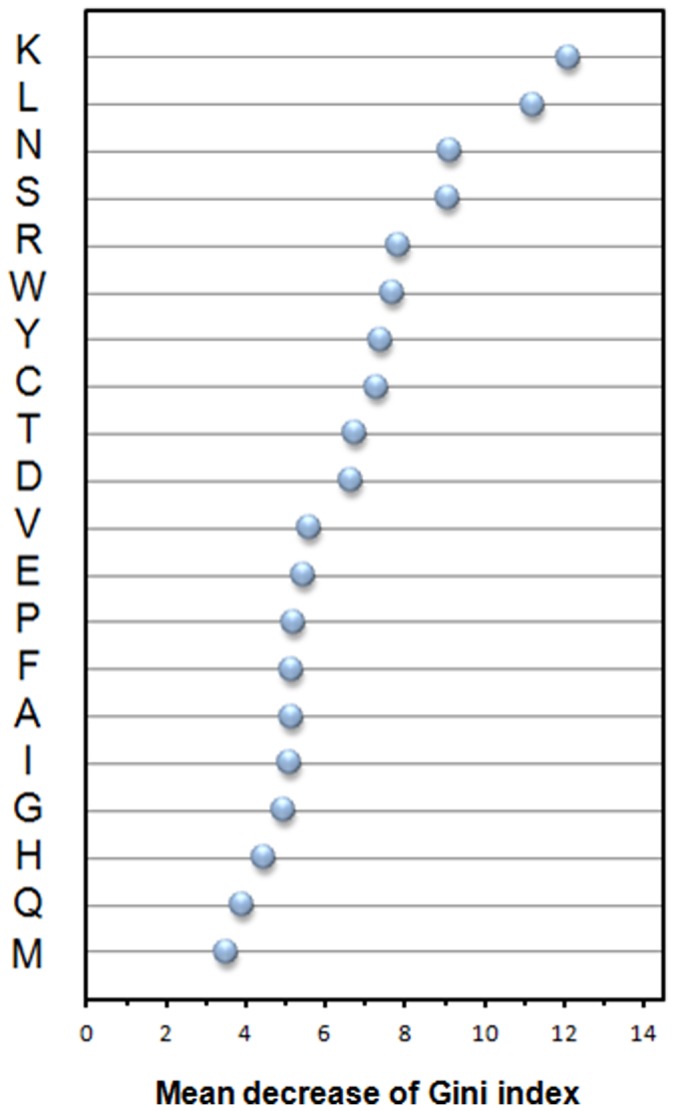
Feature importance of Amino Acid Composition (AAC). The feature with the largest value of mean decrease of Gini index (MDGI) is the most important.

The feature importance for AAC is shown in Figure 6. The feature with the largest value of MDGI is the most important. The top-four informative amino acids are lysine, leucine, asparagine and serine having an MDGI value larger than 8. There are 8 out of 10 top-ranked informative amino acids belonging to the set of polar amino acids. The polar property is especially important for predicting antibody amyloidogenesis.

**Figure 7:**
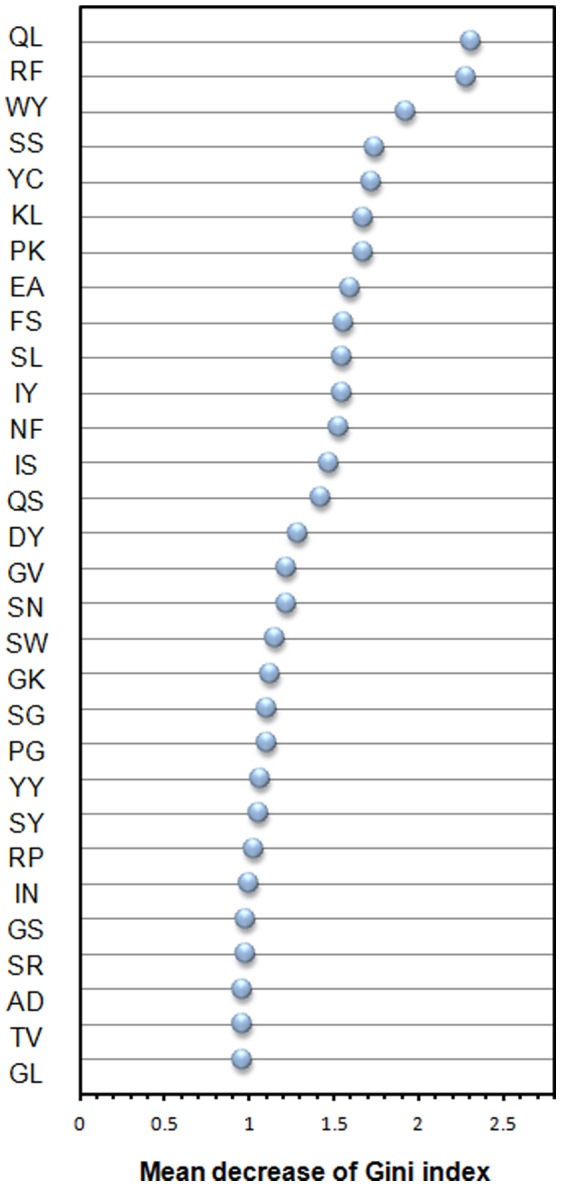
Feature importance of Dipeptide Composition (DPC). The feature with the largest value of mean decrease of Gini index (MDGI) is the most important.

**Figure 8:**
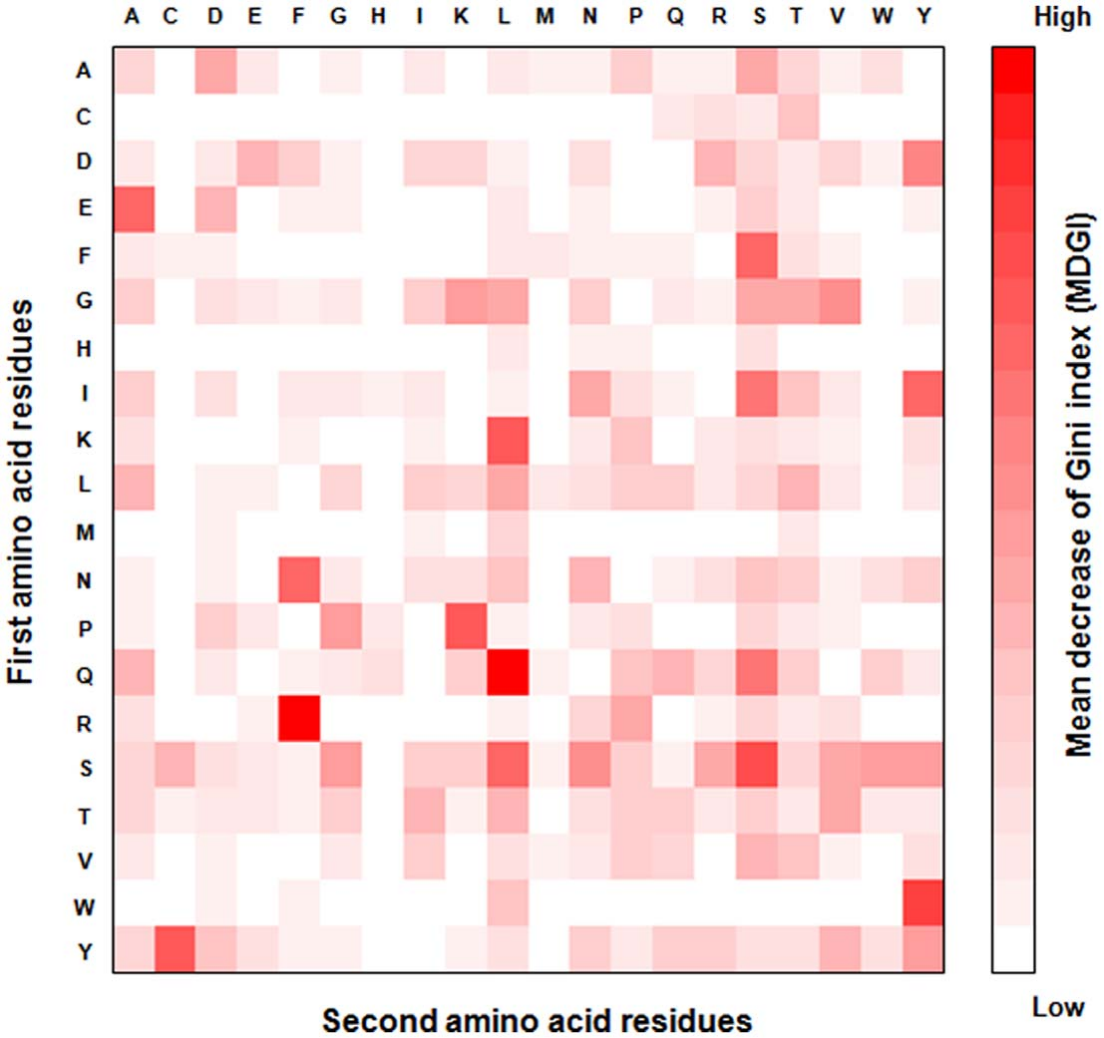
The heatmap of DPC feature importance.

The rank and heatmap of feature importance for DPC are shown in Figures 7 and 8, respectively. The top-three informative dipeptides are QL, RF and WY having the MDGI values larger than 1.9. Interestingly, 11 out of 13 top-ranked informative dipeptides consist of a polar amino acid and a nonpolar amino acid. There is no dipeptide with two nonpolar amino acids in the 30 top-ranked dipeptides. Results show that the alternating pattern of polar and nonpolar amino acids is important for antibody amyloidogenesis. The aggregation tendency of the alternating patterns is consistent with previous studies [9], [10], [11]. Another study shows that sequences with the alternating patterns are prone to form fibril structures [12].

### Feature Importance of Physicochemical Properties

**Figure 9:**
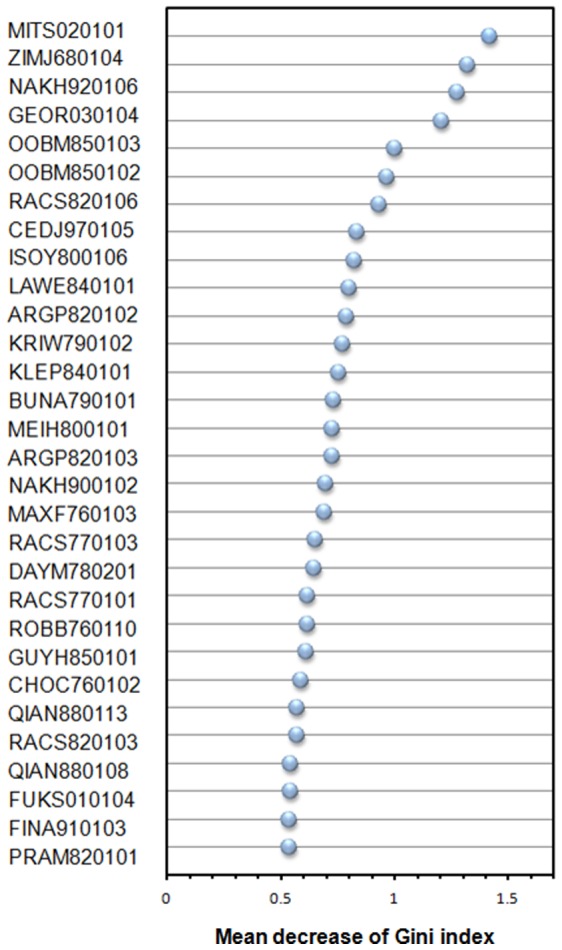
Feature importance of Physicochemical Properties (PPs). The feature with the largest value of mean decrease of Gini index (MDGI) is the most important.

We first use performance of distinguishing amyloidogenic from non-amyloidogenic to identify 30 top-ranked informative features from 531 physicochemical properties. Consequently, we classify them into 12 categorized properties using the definition of properties in AAindex and investigate correlation between the properties and amyloid fibrils aggregation. The ranks of feature importance for PPs are shown in Figure 9. The detailed information of the 30 top-ranked informative PPs is shown in Table 7. Table 8 summarizes the categorized properties and corresponding references.

**Table 7:** Top 30 informative physicochemical properties.

AAindex ID	Description
**MITS020101**	Amphiphilicity index (Mitaku et al., 2002)
**ZIMJ680104**	Isoelectric point (Zimmerman et al., 1968)
**NAKH920106**	AA composition of CYT of multi-spanning proteins (Nakashima-Nishikawa, 1992)
**GEOR030104**	Linker propensity from 3-linker dataset (George-Heringa, 2003)
**OOBM850103**	Optimized transfer energy parameter (Oobatake et al., 1985)
**OOBM850102**	Optimized propensity to form reverse turn (Oobatake et al., 1985)
**RACS820106**	Average relative fractional occurrence in ER(i) (Rackovsky-Scheraga, 1982)
**CEDJ970105**	Composition of amino acids in nuclear proteins (percent) (Cedano et al., 1997)
**ISOY800106**	Normalized relative frequency of helix end (Isogai et al., 1980)
**LAWE840101**	Transfer free energy, CHP/water (Lawson et al., 1984)
**ARGP820102**	Signal sequence helical potential (Argos et al., 1982)
**KRIW790102**	Fraction of site occupied by water (Krigbaum-Komoriya, 1979)
**KLEP840101**	Net charge (Klein et al., 1984)
**BUNA790101**	alpha-NH chemical shifts (Bundi-Wuthrich, 1979)
**MEIH800101**	Average reduced distance for C-alpha (Meirovitch et al., 1980)
**ARGP820103**	Membrane-buried preference parameters (Argos et al., 1982)
**NAKH900102**	SD of AA composition of total proteins (Nakashima et al., 1990)
**MAXF760103**	Normalized frequency of zeta R (Maxfield-Scheraga, 1976)
**RACS770103**	Side chain orientational preference (Rackovsky-Scheraga, 1977)
**DAYM780201**	Relative mutability (Dayhoff et al., 1978b)
**RACS770101**	Average reduced distance for C-alpha (Rackovsky-Scheraga, 1977)
**ROBB760110**	Information measure for middle turn (Robson-Suzuki, 1976)
**GUYH850101**	Partition energy (Guy, 1985)
**CHOC760102**	Residue accuracyessible surface area in folded protein (Chothia, 1976)
**QIAN880113**	Weights for alpha-helix at the window position of 6 (Qian-Sejnowski, 1988)
**RACS820103**	Average relative fractional occurrence in AL(i) (Rackovsky-Scheraga, 1982)
**QIAN880108**	Weights for alpha-helix at the window position of 1 (Qian-Sejnowski, 1988)
**FUKS010104**	Surface composition of amino acids in nuclear proteins (percent) (Fukuchi-Nishikawa, 2001)
**FINA910103**	Helix termination parameter at position j-2,j-1,j (Finkelstein et al., 1991)
**PRAM820101**	Intercept in regression analysis (Prabhakaran-Ponnuswamy, 1982)

**Table 8:** Categorized informative of top 30 physicochemical properties.

Categorized property	AAindex ID	References
**Amphiphilicity**	MITS020101, NAKH920106	[13], [14], [15]
**Hydrophobicity**	OOBM850103, ARGP820103, LAWE840101, CHOC760102, GUYH850101, KRIW790102, RACS770103	[16], [17], [18], [19], [20]
**Helix**	QIAN880113, QIAN880108, BUNA790101, ARGP820102, ISOY800106	[23], [24]
**Coil and Turn**	RACS820106, ROBB760110	new
**Linker propensity**	GEOR030104	new
**Reverse turn**	OOBM850102	[21], [22]
**Other structures**	RACS770101, PRAM820101, MEIH800101	new
**Isoelectric point**	ZIMJ680104, FINA910103	[25]
**Net charge**	KLEP840101	[10], [17], [26]
**Mutability**	DAYM780201	new
**Nuclear protein**	CEDJ970105, FUKS010104	new
**Uncategorized**	RACS820103, NAKH900102, MAXF760103	new

The values of MDGI for different types of features mean significantly different because each type of sequence features is independently assessed for calculating the feature importance based on 30 runs of feature importance evaluation. The importance of a feature is represented by the MDGI value which is a relative rather than absolute measurement. The mean MDGI value of one AAC feature is larger than that for one of DPC and PPs features because the influence of 1/20 change for AAC is higher than those for DPC and PPs with 1/400 and 1/531 changes, respectively. The feature importance in terms of MDGI can only be compared among the features within the same feature set.

The most important PP is the AAindex ID MITS020101 with an MDGI value of 1.416 denoting ‘amphiphilicity index’. In the analysis, two out of the 30 top-ranked informative properties are related to amphiphilicity. It is also known that the amphiphilicity can change the beta-sheet structure in the amyloid fibrils [13], and is a common property of amyloids [14], [15].

A total of seven hydrophobicity-related properties play a major role in amyloidogenesis as they occupy the major part of informative properties. If we remove one of these seven hydrophobicity-related properties in each time, the average accuracy will decrease from 79.17% to 77.32% in across-germline prediction. The importance of hydrophobicity found in this study is consistent with the results of previous bioinformatics studies on predicting amyloidogenic regions [16], [17]. The masking of hydrophobic surface inhibits protein-protein aggregation [18]. Enhancement of hydrophobicity by post-translational modification of lysine residues appeared very effective in promoting fibrillation [19]. The hydrophobic effect is a major driving force for the self-assembly of many aggregation prone polypeptides including A*β* [20]. The influence of isoelectric point and net charge on amyloid fibril formation have been reported previously [10], [17], [25], [26] and the related informative properties are ZIMJ680104, FINA910103 and KLEP840101.

The 12 structure-related properties that involved five categorizations show the important role of protein structures in antibody amyloidogenesis. The reverse turn property OOBM850102 is an important property for antiparallel structure formation of amyloid proteins [21] and amyloid-like fibril formation [22]. The helical structure of intermediates plays important roles in amyloid fibril formation [23], [24]. The property BUNA790101 representing ‘alpha-NH chemical shifts’ is also related to helix forming, which has high correlation coefficients of 0.945 and 0.902 with helix forming properties of BLAM930101 denoting ‘alpha helix propensity’ and ONEK900101 denoting ‘thermodynamic scale for the helix-forming tendencies’, respectively. The property ARGP820102 is also ranked as an important property representing the helical potential of signal sequences.

In this study, there are six newly-found categorized properties that have never been reported in previous studies, including the coil property RACS820106, the turn property ROBB760110, the linker property GEOR030104, the mutability property DAYM780201, the nuclear protein properties FUKS010104 and CEDJ970105, and the properties of other structures of RACS770101, PRAM820101 and MEIH800101.

### Ubiquitylation and Antibody Amyloidogenesis

According to the feature importance analysis for AAC, the amino acid lysine is ranked as the most important amino acid. The amino acid lysine is associated with many post-translational modifications including methylation and ubiquitylation. One of the most well-known functions of ubiquitylation is its regulatory role in protein degradations. By tagging proteins with ubiquitins, the ubiquitylated proteins will be degraded by proteasome [46]. It is interesting to know whether a protein with a larger number of ubiquitylation sites is less amyloidogenic because it tends to be degraded.

**Figure 10:**
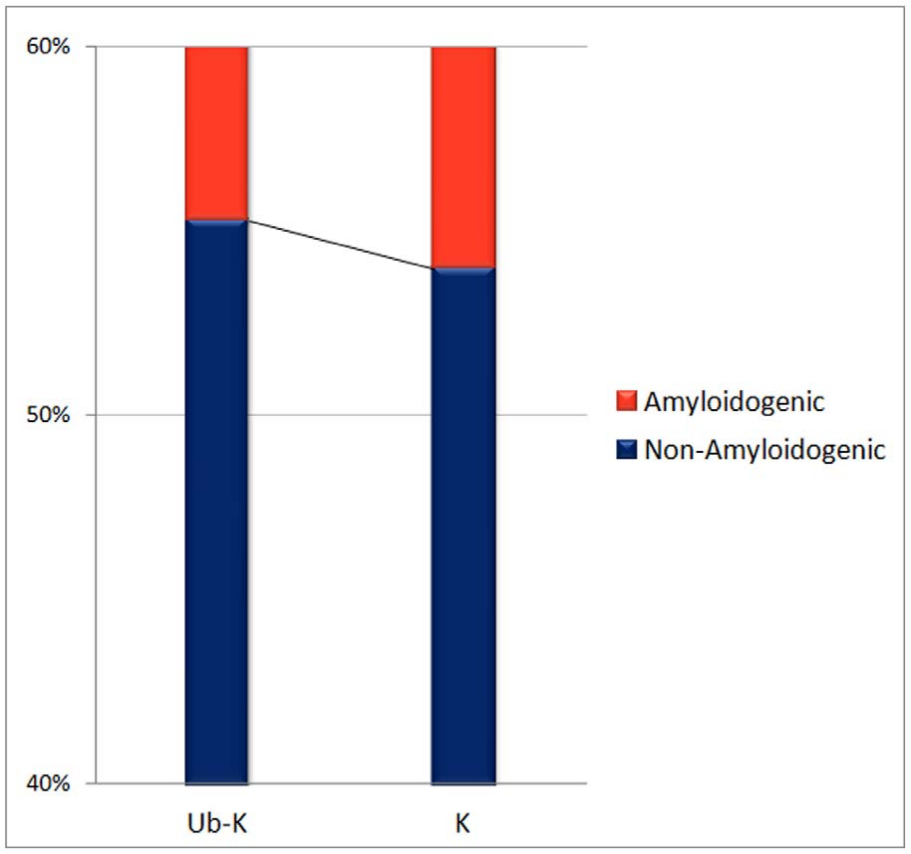
The 100% stacked column chart of the numbers of lysines (K) and putative ubiquitylated lysines (Ub-K).

To better understand the correlation between ubiquitylation sites and amyloidogenesis, the prediction method UbiPred with an accuracy of 84.44% [42] is applied to identify putative ubiquitylation sites for all sequences in the dataset AA-432. The average numbers of lysines (Ks) and putative ubiquitylated lysines (Ub-Ks) in a protein are 3.05 and 2.19 for non-amyloidogenic antibodies and 2.60 and 1.77 for amyloidogenic antibodies, respectively. The numbers of Ks and Ub-Ks for non-amyloidogenic antibodies are significantly larger than those of amyloidogenic antibodies (t-test, p-value<0.001). The ratios of the numbers of Ub-Ks to Ks are 0.717 and 0.680 for non-amyloidogenic and amyloidogenic antibodies, respectively. Figure 10 represents the fractions of the numbers of Ks and Ub-Ks in amyloidogenic and non-amyloidogenic antibodies. The difference between the numbers of Ub-Ks of amyloidogenic and non-amyloidogenic antibodies is larger than that of Ks. The results show that ubiquitylation might play important roles in determining the amyloidogenesis property.

**Figure 11:**
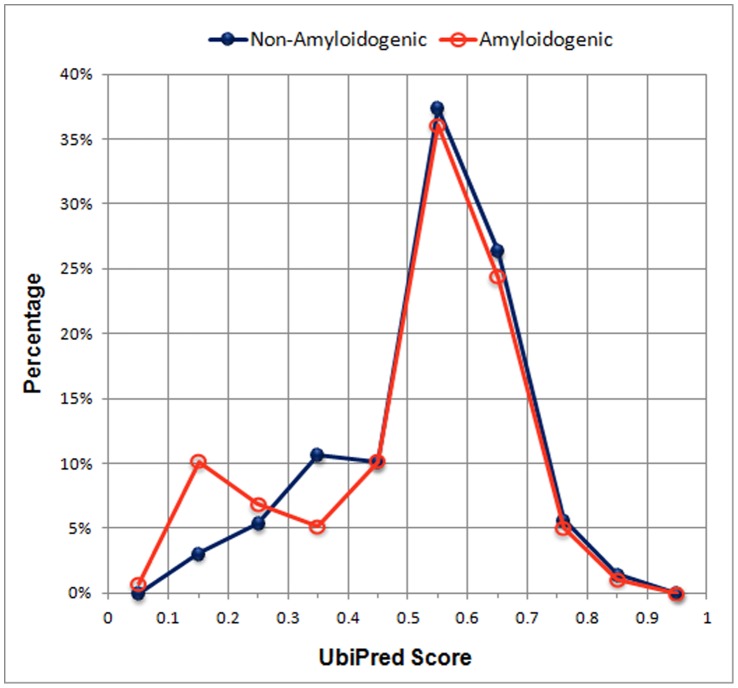
Distribution of lysines for amyloidogenic and non-amyloidogenic antibodies.

To assess the influence of Ub-Ks in the Random Forests classifiers, a total of 12 ubiquitylation features are proposed (see the Materials and Methods section). Figure 11 shows the distribution of lysines for amyloidogenic and non-amyloidogenic antibodies. Each point represents the percentage of the number of lysines whose scores predicted are in a specific range. The numbers of lysines between the amyloidogenic and non-amyloidogenic antibodies in three bins of [0, 0.1), [0.1, 0.2) and [0.3, 0.4) are significantly different (t-test, p-value<0.05).

**Figure 12:**
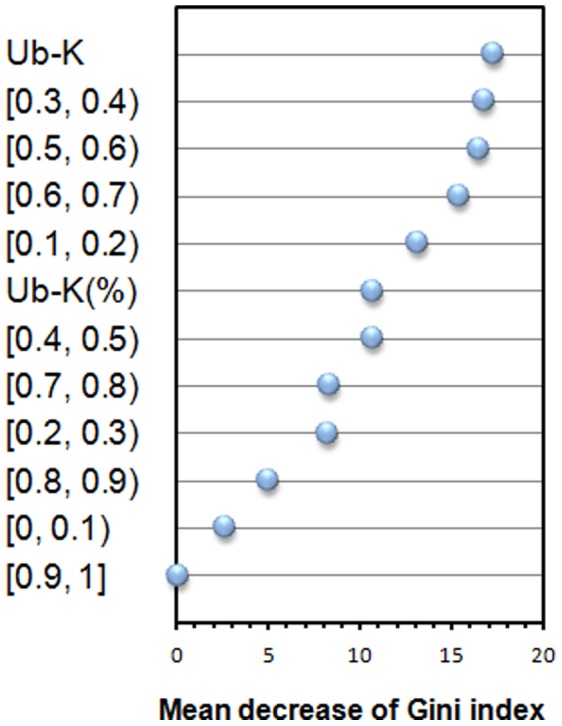
Feature importance of 12 ubiquitylation features. The feature with the largest value of mean decrease of Gini index (MDGI) is the most important.

The feature importance of the 12 features of ubiquitylation is firstly assessed by using 30 runs of the feature importance estimator of Random Forests and results are shown in Figure 12. The number of Ub-Ks is the most important feature. The ranks of the three significantly different bins of the score ranges [0, 0.1), [0.1, 0.2) and [0.3, 0.4) are 11, 5 and 2, respectively. The two features of ranges [0.5, 0.6) and [0.6, 0.7) are at ranks 3 and 4, which are not significantly different between amyloidogenic and non-amyloidogenic antibodies, shown in Figure 11. The scenario reveals that the feature importance by considering the whole set of features in prediction is not necessarily consistent with the feature significance by considering individual features using statistical analysis.

**Figure 13:**
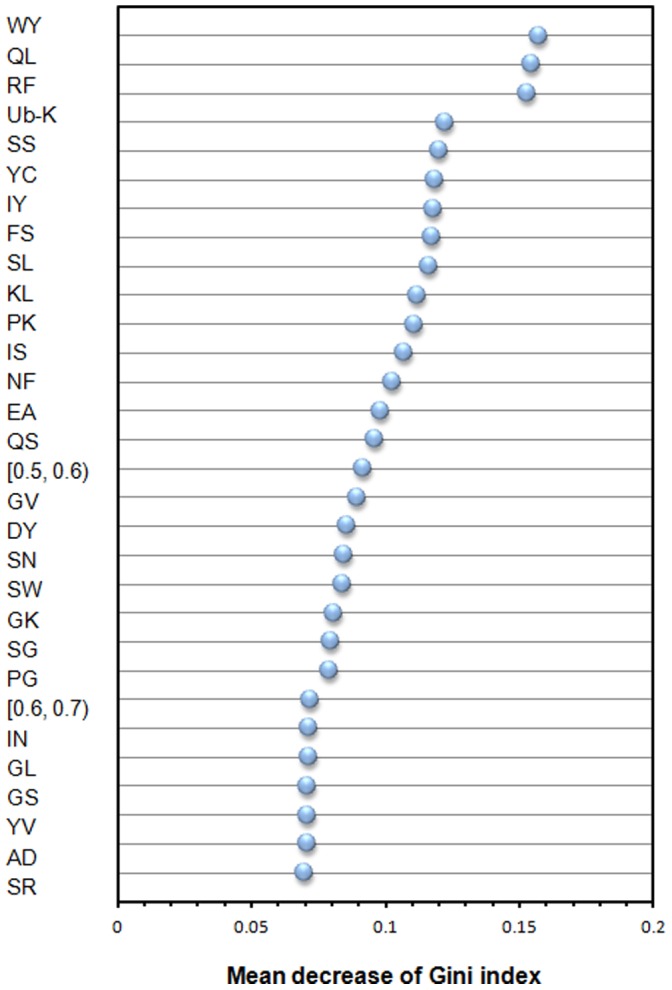
Feature importance of 12 ubiquitylation features and Dipeptide Composition (DPC). The feature with the largest value of mean decrease of Gini index (MDGI) is the most important.

Subsequently, we evaluate the feature importance using combination of the 12 features of ubiquitylation and DPC (Figure 13). In the 30 top-ranked features, the number of Ub-Ks and bins of [0.5, 0.6) and [0.6, 0.7) are at ranks 4, 16 and 24, respectively. By using Random Forests classifiers with the 412 features, the across-germline prediction accuracy and sensitivity using a Jackknife test are slightly improved from 83.33% and 85.37% to 84.03% and 86.59%, respectively, while the specificity is unchanged. For the leave-one-germline-out test using 412 features, the sensitivity is slightly improved from 78.46% to 79.27% while the accuracy is the same as that using 400 features because of the slightly decreased specificity. The distribution of lysines for amyloidogenic and non-amyloidogenic antibodies in Figure 11 might explain that the antibodies less likely to be ubiquitylated tend to be amyloidogenic, however, there might be other factors influencing the amyloidogenesis of the other antibodies. An antibody with higher number of Ub-K tends to be degraded by proteasome that is less likely to be ubiquitylated. In contrast, an antibody with lower number of Ub-K is less possible for degradation by proteasome that might accumulate to be amyloidogenic.

### Conclusions

Computational prediction of antibody amyloidogenesis can accelerate the design process of antibody humanization and save a lot of cost and time. Previous studies focused on predicting amyloidogenic potential of a given segment of proteins. However, it is desirable to develop accurate methods for predicting whether a given antibody is amyloidogenic or not.

To the best knowledge of authors, there is only one study focusing on the prediction of antibody amyloidogenesis [41]. Their methods based on accurate germline assignment by experts are not able to automatically predict antibody amyloidogenesis, especially antibodies of novel germlines. In order to develop an automated method for predicting antibody amyloidogenesis and leveraging the information across germlines, three types of germline-independent sequence features and four combinations of these feature types are assessed in terms of prediction accuracy. AbAmyloid based on dipeptide composition features performs well for predicting antibody amyloidogenesis. The within- and across-germline prediction accuracies are 83.10% and 83.33% using Jackknife tests, respectively, and the novel-germline prediction accuracy using a leave-one-germline-out test is 72.22%. The prediction server of AbAmyloid using the whole dataset AA-432 is publicly available at http://iclab.life.nctu.edu.tw/abamyloid. From simulation results, AbAmyloid is expected to be more accurate when more training samples are available.

By carefully examining the informative features obtained from the feature importance analysis of Random Forests, the dipeptide composition of a polar and a nonpolar amino acids and the property amphiphilicity are found to be of great importance for amyloidogenesis that is confirmed by published studies [13], [14], [15]. Identified informative physicochemical properties of hydrophobicity, helical structure, reverse turn, isoelectric point and net charge are found to be consistent with previous studies [16], [17], [21], [22], [23], [24], [25]. Newly identified physicochemical properties including mutability, nuclear proteins, coil, linker, turn and other structures might provide new insights into antibody amyloidogenesis.

Finally, the importance of ubiquitylation on antibody amyloidogenesis is also investigated. The addition of ubiquitylation features generated by UbiPred slightly improves the sensitivity of Random Forests classifiers. Results show that antibodies less likely to be ubiquitylated might tend to be amyloidogenic.

## Materials and Methods

### Dataset

The dataset (named AA-432) of 432 antibody light chain sequences consists of 246 amyloidogenic and 186 non-amyloidogenic derivatives of 12 germlines that is obtained from a previous study [41]. Protein sequence of germline is a non-amyloidogenic sequence, and the other sequences that are assigned in this set are derivatives of this germline. The derivative sequences are rearranged and mutated from the protein sequence of germline with only a few changes of amino acids. All the derivatives of the germlines are obtained from the National Center for Biotechnology Information (NCBI). After omitting the first five amino acid residues that are suspect to be primer-derived, the dataset is available in the supplementary information of the paper [41]. Table 9 shows the detailed numbers of amyloidogenic and non-amyloidogenic sequences for each germline.

**Table 9:** The numbers of amyloidogenic and non-amyloidogenic antibodies in the dataset AA-432.

Germline	Amyloidogenic	Non-Amyloidogenic
**J00248**	8	15
**M30446**	6	10
**X72813**	8	19
**X93620**	33	16
**X93627**	19	14
**X93632**	5	9
**X93640**	17	13
**Z22188**	34	12
**Z22191**	5	9
**Z22197**	26	17
**Z22208**	35	18
**Z73673**	50	34
**Total**	246	186

### Feature Extraction

#### Amino acid composition (AAC)

Given an antibody sequence A with length *l*, the corresponding occurrence frequency *a_i_* for the *i*-th amino acids is calculated as the following:
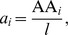
where the AA*_i_* is the count of occurrences in the sequence for the *i*-th amino acid. Finally, a 20-dimension feature vector defined as is obtained for the following analysis.

#### Dipeptide composition (DPC)

For the *i*-th dipeptide, the corresponding dipeptide frequency *d_i_* is calculated for a given antibody sequence A with length *l* as the following:
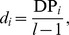
where the DP*_i_* is the count of the *i*-th dipeptide in the antibody sequence. The final vector is a 400-dimension feature vector defined as representing the occurrence frequencies of 400 dipeptides.

#### Physicochemical properties (PPs)

Physicochemical properties with good interpretability are important and useful for prediction and analysis in bioinformatics studies [42], [47], [48], [49], [50]. Analysis of correlation between properties is also investigated by factor analysis of multivariate statistical analysis on DNA binding proteins [50]. Furthermore, if the optimal feature selection from 531 PPs based on the AAindex values, a set of highly correlated PPs can be further reduced by using a representative one without significantly decreasing the prediction accuracy [48]. In this study, 544 physicochemical properties were retrieved from the amino acid indices (AAindex) database of version 9.0 [51]. The AAindex database is a collection of many published numerical indices representing physicochemical and biochemical properties of amino acids. Each physicochemical property is represented as a set of 20 numerical values for amino acids. After removing 13 physicochemical properties having the value ‘NA’ in their amino acid indices, a total of 531 physicochemical properties are used for the following studies.

The encoding method for an antibody sequence consists of two steps. The first step is to convert an antibody sequence to 531 index vectors. Given an antibody sequence A with length *l*, 531 index vectors, *p* = 1, …, 531, for 531 physicochemical properties are obtained by substituting the amino acids with corresponding index values. The second step constructs the final feature vector for representing an antibody sequence. The feature vector is defined as, where *v_p_* is the averaged value of elements in X*_p_*.

#### Ubiquitylation features

A total of 12 ubiquitylation features are proposed for assessing the influence of Ub-Ks in the Random Forests classifiers, calculated as follows. First, for each lysine (K) in the sequence, the prediction scores ranging from 0 to 1 obtained from UbiPred [42] representing the ubiquitylation probabilities are assigned to ten bins of equal ranges. Therefore, the ten percentage values representing the numbers of lysines in ten score ranges are used as features of ubiquitylation. Second, two additional features are the number of putative ubiquitylated lysines (Ub-Ks) and the ratio of the numbers of putative ubiquitylated lysines (Ub-Ks (%)) to Ks.

### Random Forests

The Random Forests classifier based on a large ensemble of decision trees is an extensively used ensemble learning method [52]. The Random Forests classifier improves prediction performances of classification and regression trees (CART, [53]) by growing many weak CART trees. Every tree is built by using a fixed number of randomly selected features for tree splitting and is based on a bootstrap sample of the whole training dataset. The advantages of the Random Forests classifier include less overfitting problems [54], [55]. The property of avoiding overfitting problems is especially important when analyzing a small dataset in this study. It shows high predictive accuracy and is applicable even in high-dimensional problems with highly correlated variables, a situation which often occurs in bioinformatics [56]. Additionally, Random Forests is good in handling redundant features that is reported previously [57], [58]. In this study, 100 trees are utilized to construct a Random Forests classifier, and the number of selected features is set to a default value of the square root of the total number of features [52].

### Feature Importance

The Random Forests classifier is useful for evaluating feature importance by using out-of-bag (OOB) data. In the training of a Random Forests classifier, two-third of a training dataset is applied to build the classifier and the other one-third (OOB data) of the training dataset is utilized to evaluate the performance of the classifier. To evaluate the importance for each feature X*_i_*, the values of feature X*_i_* in OOB data are randomly permutated and feature importance for X*_i_* can be evaluated by measuring the decrease of prediction performance of the permutated OOB data. The performance measurement can be accuracy or Gini index. The Gini index is a measure of impurity representing the ability of a potential split for separating the samples of two classes that can be defined as 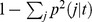, where 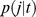 denotes the estimated class probabilities for a node *t* in a decision tree and class 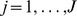 [52]. In this study, 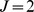 denotes the amyloidogenic and non-amyloidogenic. The mean decrease of Gini index (MDGI) is utilized to estimate feature importance because MDGI is suggested to be more robust than the mean decrease of accuracy [45]. The feature with the largest value of MDGI is the most important feature because it contributes most to prediction performances.

### Performance Evaluation

Three measurements are used to evaluate the prediction performance of the proposed methods using both Jackknife test and leave-one-germline-out test on the constructed datasets, namely sensitivity (SEN), specificity (SPE) and accuracy (ACC) as defined in the follows: SEN = TP/(TP+FN), SPE = TN/(TN+FP) and ACC = ((TP+TN)/(TP+FN+TN+FP))*100%, where the TP, TN, FP and FN are the numbers of true positive, true negative, false positive and false negative, respectively.
